# The effectiveness of gabapentin and gabapentin/montelukast combination compared with dextromethorphan in the improvement of COVID‐19‐ related cough: A randomized, controlled clinical trial

**DOI:** 10.1111/crj.13529

**Published:** 2022-07-31

**Authors:** Rasool Soltani, Sara Nasirharandi, Farzin Khorvash, Maryam Nasirian, Kian Dolatshahi, Atousa Hakamifard

**Affiliations:** ^1^ Infectious Diseases and Tropical Medicine Research Center Isfahan University of Medical Sciences Isfahan Iran; ^2^ Department of Clinical Pharmacy and Pharmacy Practice, School of Pharmacy and Pharmaceutical Sciences Isfahan University of Medical Sciences Isfahan Iran; ^3^ Department of Infectious Diseases, School of Medicine Isfahan University of Medical Sciences Isfahan Iran; ^4^ Nosocomial Infection Research Center Isfahan University of Medical Sciences Isfahan Iran; ^5^ Department of Biostatistics and Epidemiology, School of Health Isfahan University of Medical Sciences Isfahan Iran; ^6^ Infectious Diseases and Tropical Medicine Research Center Shahid Beheshti University of Medical Sciences Tehran Iran

**Keywords:** allergy, antitussive agents, cough, hypersensitivity, pneumonia, SARS‐CoV‐2

## Abstract

**Introduction:**

Cough is one of the most common presenting symptoms of COVID‐19, which can persist for weeks or months.

**Objective:**

The goal of this study was to evaluate the effectiveness of gabapentin (GBT) alone and in combination with montelukast (MTL) for improving cough.

**Methods:**

In this open‐label randomized controlled clinical trial, eligible cases were patients hospitalized with moderate to severe COVID‐19 who had cough with a Breathlessness, Cough, and Sputum Scale (BCSS) score of at least 2 based on its cough subscale. The participants were randomly assigned to three groups including two experimental groups and one control group. The first and second experimental groups received GBT and GBT/MTL, respectively, whereas the control group received dextromethorphan (DXM). Treatment duration was 5 days in all groups. Before and after the interventions, the severity of cough was evaluated using BCSS scale and Visual Analog Scale (VAS).

**Results:**

A total of 180 patients were included; GPT, GPT/MTL, and DXM consisted of 76, 51, and 53 patients, respectively. There was no significant difference between the three groups in terms of age, gender, and comorbidities (*P* > 0.05). Regarding BCSS and VAS scores, there was significant reduction from the baseline values in all groups (*P* < 0.0001), with the change rate being significantly higher in DXM group. The amount of reduction of BCSS in the GPT/MTL group was significantly more than the GPT group, whereas there was no significant difference between the two groups regarding VAS score. Although the duration of hospitalization differed between the groups with the GPT/MTL group having the shortest duration, the difference was statistically significant only between the GPT and GPT/MTL groups (*P* < 0.0001).

**Conclusion:**

GPT, both alone and in combination with MTL, improves cough frequency and severity in hospitalized patients with COVID‐19, with the combination being more efficacious. This regimen may be useful in patients who cannot tolerate opioids.

## Graphical abstract

The evaluation of effectiveness of gabapentin alone and in combination with montelukast for improving cough.

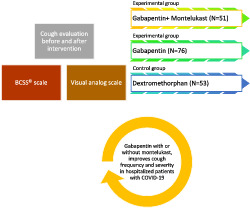

## 1 INTRODUCTION

At the end of December 2019, there were several unaccounted cases of pneumonia in Wuhan, China. Within days, the cause of this pneumonia was identified as the novel coronavirus, later known as SARS‐CoV‐2 with its related disease being named COVID‐19.^1^ SARS‐CoV‐2 spread rapidly to China and then to the rest of the world, so the World Health Organization (WHO) declared the pandemic on 11 March 2020.^2^ Globally, there have been more than 364 million of confirmed cases of COVID‐19, including more than 5 million deaths, reported to WHO.^3^ COVID‐19 has some common symptoms including fever, cough, and fatigue. This study focuses on the cough with a prevalence of 67.8%, starting 1 day after the disease onset and lasting up to 19 days on average.^4^

Coughing is a reflex that activates the vagus nerve, which feeds the brain stem at the solitary and trigeminal spinal nucleus.^5^ Immune cells infiltrate the peripheral nervous system and induce cough by inflammatory reactions.^6^ Due to the above mechanisms, cytokines (IL‐1β, TNF, and interferons) are released from the innate immune response affecting peripheral neurons.^7^ Considering the high prevalence of cough in COVID‐19, it is necessary to find effective treatments, including medications, to control this bothersome symptom.

Gabapentin (GPT), a cyclic gamma‐aminobutyric acid (GABA) analog and antiepileptic drug, has been shown to effectively improve unexplained chronic cough.^8^ It can also decrease the frequency and severity of idiopathic and chronic coughs in cancer patients, particularly those with lung cancer.^9^

It seems that cysteinyl leukotrienes have an important role in postinfectious cough. It has been shown that rhinovirus infection is associated with the increased expression of 5‐lipoxygenase, which is involved in the production of cysteinyl leukotrienes.^10^ Furthermore, increased concentrations of cysteinyl leukotriene has been reported in the nasal secretions of adults during several viral infections.^11^ Montelukast (MTL) is a drug effective for managing the symptoms of asthma and allergic rhinitis.^12^ It binds to cysteinyl leukotriene receptors and by inhibiting the activity of leukotriene CysLT1 (LTD4) prevents the inflammatory cascade that causes these disorders.^12^ Therefore, considering the role of LTD4 in postinfectious cough, montelukast may have an antitussive effect in COVID‐19 associated cough.

Due to the novelty of COVID‐19, much research is now being done to assess the impact of various drugs on this disease and its complications. Based on the potential antitussive effects of GPT and MTL, the present clinical study was designed to investigate the possible positive impact of these two drugs on cough in hospitalized COVID‐19 patients.

## 2 MATERIALS AND METHODS

This open‐label randomized controlled clinical trial was conducted between April and May 2020 at Al‐Zahra hospital of Isfahan, Iran, affiliated to Isfahan University of Medical Sciences (IUMS). All included patients studied and signed the written informed consent form. Also, the study was approved by the ethics committee of IUMS (ethics code: IR.MUI.MED.REC.1399.952).

### 2.1 Patients

Study population was selected from the hospitalized patients diagnosed with COVID‐19. They were eligible for the study if they were at least 18 years old, were hospitalized with the diagnosis of moderate to severe COVID‐19 by a positive nasopharyngeal SARS‐CoV‐2 PCR test and/or a lung CT‐scan suggestive of COVID‐19, and had cough with a Breathlessness, Cough, and Sputum Scale (BCSS) score of at least 2 based on its cough subscale.

Patients who were intubated, allergic to each of the study drugs, unconscious, taking any ACEI (angiotensin converting enzyme inhibitor), SSRIs (selective serotonin reuptake inhibitor), SNRI (serotonin norepinephrine reuptake inhibitor) or antitussive agents before admission, had a chronic respiratory disease or allergy, and had oral intake intolerance, were all excluded from the study.

### 2.2 Interventions and assessments

Upon inclusion of each patient, baseline information, including demographic characteristics and comorbidities, were recorded for him/her. The included participants were randomly assigned to three groups including two experimental groups and one control group. The samples were determined and assigned to three groups by random number table. Even numbers considered for the dextromethorphan (DXM) group, odd numbers for the gabapentin (GPT) group, and zero for the montelukast (MTL)/GPT group.

Upon admission, the first and second experimental groups received 300‐mg oral capsules of gabapentin (Raha, Iran) every 8 h (GPT group) and 300‐mg oral capsules of gabapentin every 8 h and 10‐mg oral tablets of montelukast (Dr. Abidi, Iran) every evening (GPT/MTL), respectively, for 5 days, whereas the control group received 10 ml (30 mg) oral syrup of dextromethorphan (Pursina, Iran) every 8 h (DXM group), for 5 days. Before and after the interventions, the severity of cough was evaluated using BCSS© scale and Visual Analog Scale (VAS). BCSS is an outcome measure scale for evaluation of symptoms in patients with chronic obstructive pulmonary disease (COPD). It rates the severity of the three symptoms, each on a 5‐point scale, so that higher scores represent more severe symptoms.^13^ However, in this study, cough subscale with the question “How was your cough today?” and the following scores according to the patient's response was used: 0 = no cough—unaware of coughing; 1 = rare—cough now and then; 2 = occasional—less than hourly; 3 = frequent—one or more times an hour; 4 = almost constant—never free of cough or need to cough. Using VAS, the patients rated their cough severity from 0 (no cough) to 10 (the most severe cough).

The primary outcome measures were the change of BCSS and VAS scores from the baseline values, whereas the secondary outcome variable was the duration of hospitalization.

### 2.3 Statistical analysis

The quantitative variables were presented as the mean and standard deviation (SD) or median and interquartile range (IQR), whereas the categorical variables were presented as frequency and percentage. The normality of data were evaluated by Kolmogorov–Smirnov and Shapiro–Wilk (when *n* < 50) tests. Except for hospitalization duration, all other numerical variables had a normal distribution (*P* > 0.05); so, hospitalization duration was transformed by natural logarithm due to positive skewness. Chi‐square or Fisher's exact test were used to compare categorical variables between the three groups, whereas one‐way analysis of variance (ANOVA) (with Tukey or Dunnett C for post hoc analysis) was applied to compare continuous variables between the groups. Analysis of covariance (ANCOVA) test was also used to control the group differences before the intervention on the outcomes. Data were analyzed in SPSS software (version 21), considering 0.05 for statistical significance.

## 3 RESULTS

### 3.1 Patients

**FIGURE 1:**
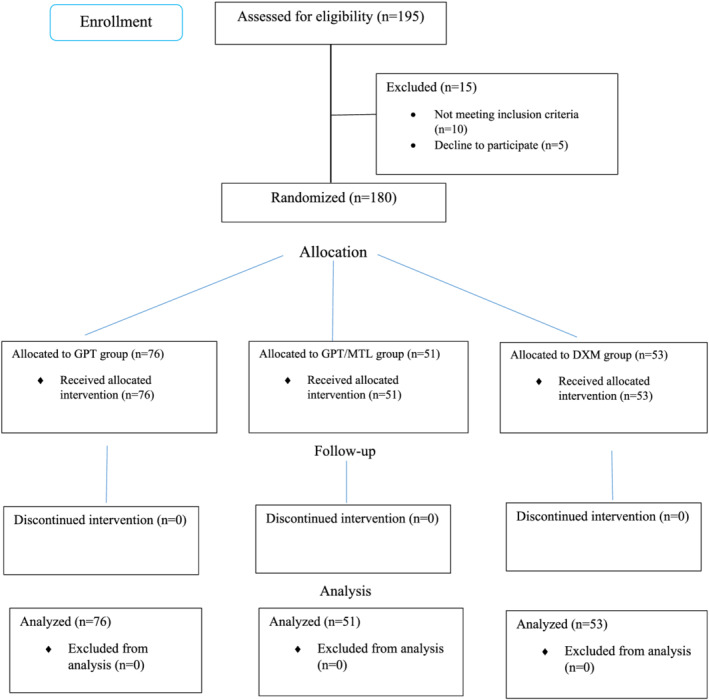
The study's CONSORT flowchart

The study included 180 patients (Figure 1), with a mean (SD) age of 56.78 (14.48); 101 (56.11%) of the patients were men. GPT, GPT/MTL, and DXM comprised 76, 51, and 53 patients, respectively. As shown in Table 1, there was no significant difference between the three groups in terms of age, gender, and comorbidities (*P* > 0.05).

**TABLE 1:** Entry characteristics

Variable	GPT *n* = 76	GPT/MTL *n* = 51	DXM *n* = 53	*P* value*
Age [years; mean (SD)]	57.33 (13.99)	54.88 (15.64)	57.83 (14.09)	0.534
Gender [*n* (%)]				
Male	44 (57.9)	27 (52.9)	30 (56.6)	0.856
Female	32 (42.1)	24 (47.1)	23 (43.4)	
Comorbidity [*n* (%)]				
Diabetes	10 (13.2)	10 (19.6)	9 (17.0)	0.612
Hyperlipidemia	5 (6.6)	3 (5.9)	7 (13.2)	0.308
Hypertension	12 (15.8)	14 (27.5)	14 (26.4)	0.205

* Chi‐square or exact test for categorical variables, and ANOVA or Kruskal–Wallis for continuous variables.

### 3.2 Assessments

The treatment groups were unbalanced in terms of baseline severity. Table 2 comparatively shows the effects of the three interventions on the evaluated outcome variables. As shown, regarding BCSS and VAS scores, there was significant reduction from the baseline values in all groups, with the change rate (the amount of reduction) being significantly higher in DXM group than the other two groups. Furthermore, as shown, according to the post hoc analysis, the amount of reduction of BCSS in GPT/MTL group was significantly more than GPT group, whereas there was no significant difference between the two groups regarding VAS score.

**TABLE 2:** Comparatively shows the effects of the three interventions on the evaluated outcome variables

Variable	Group				*P* value_1_	*P* value_2_	*P* value_3_	*P* value_4_
	Time	GPT *n* = 76	GPT/MTL *n* = 51	DXM *n* = 53				
BCSS [mean (SD)]	Baseline	3.96 (0.85)	3.29 (0.50)	3.23 (0.46)	<0.0001	<0.0001	<0.0001	0.864
	End	2.49 (1.03)	1.33 (0.86)	0.34 (0.62)	<0.0001	<0.0001	<0.0001	<0.0001
	Difference	1.47 (0.81)	1.96 (0.69)	2.89 (0.32)	<0.0001	<0.0001	<0.0001	<0.0001
	*P* value_5_	<0.0001	<0.0001	<0.0001	‐	‐	‐	‐
VAS [mean (SD)]	Baseline	3.12 (1.08)	2.75 (0.99)	2.62 (0.92)	0.016	0.107	0.019	0.812
	End	1.68 (0.59)	0.94 (0.75)	0.25 (0.58)	<0.0001	<0.0001	<0.0001	<0.0001
	Difference	1.43 (1.13)	1.80 (1.11)	2.37 (0.82)	<0.0001	0.127	<0.0001	0.016
	*P* value_5_	<0.0001	<0.0001	<0.0001	‐	‐	‐	‐
Hospitalization duration (median [IQR])	During the study	10 (7)	8 (3)	9 (5)	<0.000^*^	<0.0001	0.079	0.149

*Note*: *P* value_1_: Comparison of the three groups (ANOVA or ANCOVA); *P* value_2_: Comparison of GPT and GPT/MTL groups (Tukey post hoc test); *P* value_3_: Comparison of GPT and DXM groups (Tukey post hoc test); *P* value_4_: Comparison of GPT/MTL and DXM groups (Tukey post hoc test); *P* value_5_: Comparison of end and baseline values within each group (paired‐samples *t* test).

* Distribution was not normal and it was normalized by natural logarithm due to positive skewness (1.99).

As shown in Table 2, although the duration of hospitalization differed between the groups with GPT/MTL group having the shortest duration, the difference was statistically significant only between GPT and GPT/MTL groups. In terms of adverse effect, we did not see any side effects in any of the treatment groups.

## 4 DISCUSSION

In the present study, both experimental interventions GPT and GPT/MTL groups showed significant reduction in the severity of COVID‐19‐induced cough; however, the observed effects was significantly less than DXT as a standard antitussive drug. Furthermore, the combination of GPT and MTL showed more improvement in cough compared with GPT alone. Overall, these results show the effectiveness of both GPT and GPT/MTL regimens in the improvement of cough as a common bothersome symptom of COVID‐19.

To the best of our knowledge and according to our searches, this is the first study evaluating GPT and MTL for COVID‐19‐induced cough. However, there are a few studies for these drugs in other types of cough. In the study of Ryan et al. on patients with refractory chronic cough, GPT resulted in significant improvement in the scores of cough severity and quality of life.^14^ However, in this study, the applied dose of GPT (the maximum tolerable daily dose of 1800 mg) was twice that in our study (900 mg daily). Therefore, according to our results, it seems that gabapentin is effective in acute cough with lower doses than that that has been suggested for refractory chronic cough. Furthermore, our results show the potential antitussive effect of GPT with short duration of use, since the duration of intervention in our study was 5 days compared with 10 weeks in the mentioned study of chronic cough. In another study conducted by Dong et al., GPT, with the dose of 900 mg daily, resulted in significant improvement of suspected refractory gastro‐esophageal reflux‐induced chronic cough.^15^ This is consistent with our results regarding the applied effective dose. The antitussive effect of GPT might be related to its central effects, including inhibitory action on the release of substance P (an excitatory neurotransmitter with a role in cough induction), as this drug is a lipophilic agent with high CNS penetration.^16^ The study of Ryan et al. showed that GPT has no effect on peripheral cough reflex sensitivity to capsaicin. According to the authors, this suggests that GPT do not act by influencing the peripheral sensitization.^14^

There are very few studies on the antitussive effects of MTL in diseases other than cough variant asthma. In the study of Wang et al., MTL (10 mg/day for 2 to 4 weeks) had no significant effect on postinfectious cough compared with placebo.^17^ On the other hand, the study of Stelmach et al. showed the positive effects of MTL on the symptoms of patients with cystic fibrosis (CF) including cough.^18^ Therefore, it seems that the antitussive effects of MTL depends on the causative disease and concurrent drug, as its positive effect on cough in our study was seen for its combination with GPT. Cysteinyl leukotrienes may have a role in COVID‐19 induced cough as a viral disease. Increased expression of 5‐lipoxygenase, the enzyme responsible for production of cysteinyl leukotrienes, has been shown in rhinovirus infection.^10^ Also, high concentrations of cysteinyl leukotrienes in the nasal secretions of adults with respiratory syncytial virus (RSV), rhinovirus, and influenza A virus infections have been detected.^11^ Moreover, it has been suggested that cysteinyl leukotrienes have a role in airway inflammation induced by the viruses.^19^ These issues somewhat explain the positive effects of MTL in the suppression of cough in our study.

Overall, according to our results, both GPT and GPT/MTL could be appropriate alternatives for DXM for improvement of cough in patients with COVID‐19. Considering some limitations of DXM, including its interactions with serotonergic drugs leading to serotonin syndrome^20^ and potential of abuse because of its euphoric, hallucinogenic, and dissociative properties,^21^ these drug regimens could be considered in patients with any contraindications to DXM. However, more studies with larger sample size are required to confirm our observed effects.

The main limitations of our study were lack of placebo, small sample size, short duration of intervention, not using Leicester Cough Questionnaire and inconsistent number of patients in the study groups (because of randomization method). However, this work is the first study showing the potential benefits of GPT and MTL for improvement of cough that is one of the most troublesome symptoms of COVID‐19.

## 5 CONCLUSION

GPT, both alone and in combination with MTL, improves cough frequency and severity in hospitalized patients with COVID‐19, with the combination being more efficacious. This regimen may be useful in patients who cannot tolerate opioids. More studies are needed.

## CONFLICT OF INTEREST

The authors declare no conflict of interest.

## AUTHOR CONTRIBUTIONS

Rasool Soltani and Atousa Hakamifard designed the study, Sara Nasirharandi and Farzin Khorvash performed the study. Sara Nasirharandi collected the data. Maryam Nasirian analyzed the data. Rasool Soltani wrote the first draft of the manuscript. All authors have read and approved the final manuscript.

## ETHICS STATEMENT

The study was approved by the ethics committee of Isfahan University of Medical Sciences (ethics code: IR.MUI.MED.REC.1399.952).

## Data Availability

The data that support the findings of this study are available from the corresponding author upon reasonable request.

## References

[1] He F , Deng Y , Li W . Coronavirus disease 2019: what we know? J med Virol. 2020;92(7):719‐725. doi:10.1002/jmv.25766 32170865 PMC7228340

[2] Shah SGS , Farrow A . A commentary on “World Health Organization declares global emergency: a review of the 2019 novel coronavirus (COVID‐19)”. Int J Surg. 2020;76:128‐129. doi:10.1016/j.ijsu.2020.03.001 32169574 PMC7128929

[3] https://covid19.who.int (Accessed: 29/1/2022).

[4] Guan W , Ni Z , Hu Y , et al. Clinical characteristics of coronavirus disease 2019 in China. N Engl J med. 2020;382(18):1708‐1720. doi:10.1056/NEJMoa2002032 32109013 PMC7092819

[5] Canning BJ , Chang AB , Bolser DC . Anatomy and neurophysiology of cough: CHEST guideline and expert panel report. Chest. 2014;146(6):1633‐1648. doi:10.1378/chest.14-1481 25188530 PMC4251621

[6] Verzele NAJ , Chua BY , Law CW . The impact of influenza pulmonary infection and inflammation on vagal bronchopulmonary sensory neurons. FASEB j. 2021;35(3):e21320. doi:10.1096/fj.202001509R 33660333

[7] Chiu IM , von Hehn CA , Woolf CJ . Neurogenic inflammation and the peripheral nervous system in host defense and immunopathology. Nat Neurosci. 2012;15(8):1063‐1067. doi:10.1038/nn.3144 22837035 PMC3520068

[8] Gibson P , Wang G , McGarvey L , Vertigan AE , Altman KW , Birring SS . Treatment of unexplained chronic cough: CHEST guideline and expert panel report. Chest. 2016;149(1):27‐44. doi:10.1378/chest.15-1496 26426314 PMC5831652

[9] Razzak R , Waldfogel JM , Doberman DJ , Feliciano JL , Smith TJ . Gabapentin for cough in cancer. J Pain Palliat Care Pharmacother. 2017;31(3–4):195‐197. doi:10.1080/15360288.2017.1420120 29381133

[10] Seymour ML , Gilby N , Bardin PG , et al. Rhinovirus infection increases 5‐lipoxygenase and cyclooxygenase‐2 in bronchial biopsy specimens from nonatopic subjects. J Infect Dis. 2002;185(4):540‐544. doi:10.1086/338570 11865407

[11] Gentile DA , Fireman P , Skoner DP . Elevations of local leukotriene C4 levels during viral upper respiratory tract infections. Ann Allergy Asthma Immunol. 2003;91(3):270‐274. doi:10.1016/S1081-1206(10)63529-6 14533659

[12] Trinh HKT , Lee SH , Cao TBT , Park HS . Asthma pharmacotherapy: an update on leukotriene treatments. Expert Rev Respir med. 2019;13(12):1169‐1178. doi:10.1080/17476348.2019.1670640 31544544

[13] Leidy NK , Schmier JK , Jones MK , Lloyd J , Rocchiccioli K . Evaluating symptoms in chronic obstructive pulmonary disease: validation of the Breathlessness, Cough and Sputum Scale ©. Respir med. 2003;97(Suppl A):S59‐S70. doi:10.1016/S0954-6111(03)80016-1 12564612

[14] Ryan NM , Birring SS , Gibson PG . Gabapentin for refractory chronic cough: a randomised, double‐blind, placebo‐controlled trial. Lancet. 2012;380(9853):1583‐1589. doi:10.1016/S0140-6736(12)60776-4 22951084

[15] Dong R , Xu X , Yu L , et al. Randomised clinical trial: gabapentin vs baclofen in the treatment of suspected refractory gastro‐oesophageal reflux‐induced chronic cough. Aliment Pharmacol Ther. 2019;49(6):714‐722. doi:10.1111/apt.15169 30740748

[16] Kimos P , Biggs C , Mah J , et al. Analgesic action of gabapentin on chronic pain in the masticatory muscles: a randomized controlled trial. Pain. 2007;127(1):151‐160. doi:10.1016/j.pain.2006.08.028 17030096

[17] Wang K , Birring SS , Taylor K , et al. Montelukast for postinfectious cough in adults: a double‐blind randomised placebo‐controlled trial. Lancet Respir med. 2014;2(1):35‐43. doi:10.1016/S2213-2600(13)70245-5 24461900

[18] Stelmach I , Korzeniewska A , Stelmach W , Majak P , Grzelewski T , Jerzynska J . Effects of montelukast treatment on clinical and inflammatory variables in patients with cystic fibrosis. Ann Allergy Asthma Immunol. 2005;95(4):372‐380. doi:10.1016/S1081-1206(10)61156-8 16279568

[19] Matsuse H , Hirose H , Tsuchida T , et al. Effects of respiratory syncytial virus infection on dendritic cells and cysteinyl leukotrienes in lung tissues of a murine model of asthma. Allergol Int. 2007;56(2):165‐169. doi:10.2332/allergolint.O-06-476 17460444

[20] Baldo BA . Opioid analgesic drugs and serotonin toxicity (syndrome): mechanisms, animal models, and links to clinical effects. Arch Toxicol. 2018 Aug;92(8):2457‐2473. doi:10.1007/s00204-018-2244-6 29916050

[21] Ahmed G , Saleem MD , Naim H . How many deaths before we put cough syrups behind the counter? Perspect Public Health. 2014 Nov;134(6):309. doi:10.1177/1757913914551914 25485349

